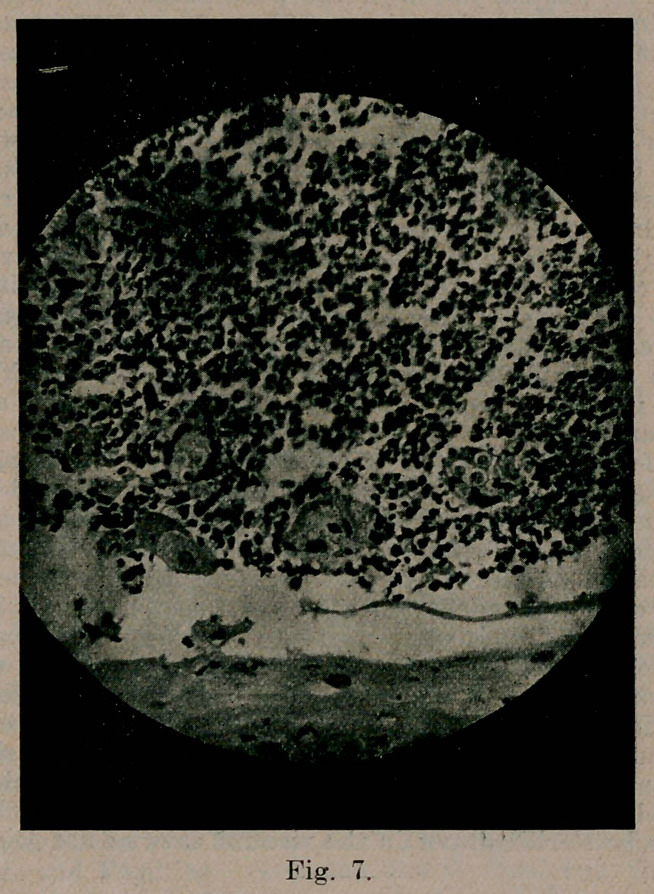# Six Cases of Blastomycetic Infection of Eyelids Reported in Memphis

**Published:** 1916-05

**Authors:** 


**Six Cases of Blastomycetic Infection of Eyelids Reported in Memphis.** Robert Fagin, Memphis, Ophthalmoscope, Sept.

Fig. 1.

1915, Memphis Med. Monthly, Dec. 1915. (Cuts by courtesy of Editor of latter). This report was the thesis for the degree of Doctor of Ophthalmology, Univ. of Col., 1915. Due credit is given to the physicians whose cases are described, one case being under the author's personal care, throughout. 5 cases were negroes.

Fig. 2.

Case 1 (see Fig. 1) began in summer of 1905, treated a little over a year later by large doses of iodides and X-ray. Ectropion resulted, plastic operation with skin graft from patient's arm. Cure, shown by picture made April 1915.

Case 2. (See Figs. 2 and 3). Began 1900 in left eye, first seen 1908, has recently involved right side.

Case 3. (not illustrated) Began 1908, first seen 1910, involved both right lids.

Case 4. (See Fig. 4). Began 1912, first lesion on nose.

Fig. 3.

Fig. 4.

Fig. 5.

Fig. 6

Picture made April 1914. This is the only case in a white person reported from Memphis.

Case 5. First seen and photographed March 1915. Initial lesion on outer canthus of right eye. This is the only case in the series in which lesions at a distance, were noted, namely on the arms and one leg.

Fig. 7.

Case 6. (See Figs. 6 and 7). Began 1911, first seen and photographed Nov. 1913. April 1914, after iodid and X-ray treatment, the case seemed practically healed with little ectropion. June 1, after a hearty meal, he became unconscious and soon died. No necropsy. Unilateral paralysis observed and suspicion of blastomycetic brain lesion.

## Figures and Tables

**Fig. 1. f1:**
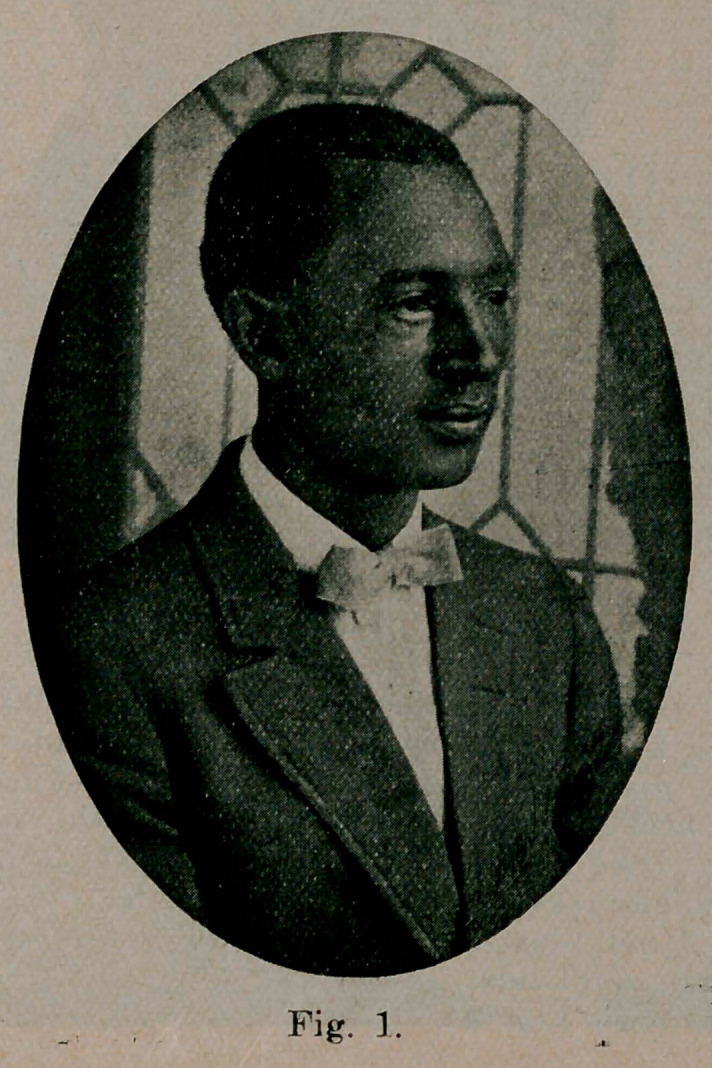


**Fig. 2. f2:**
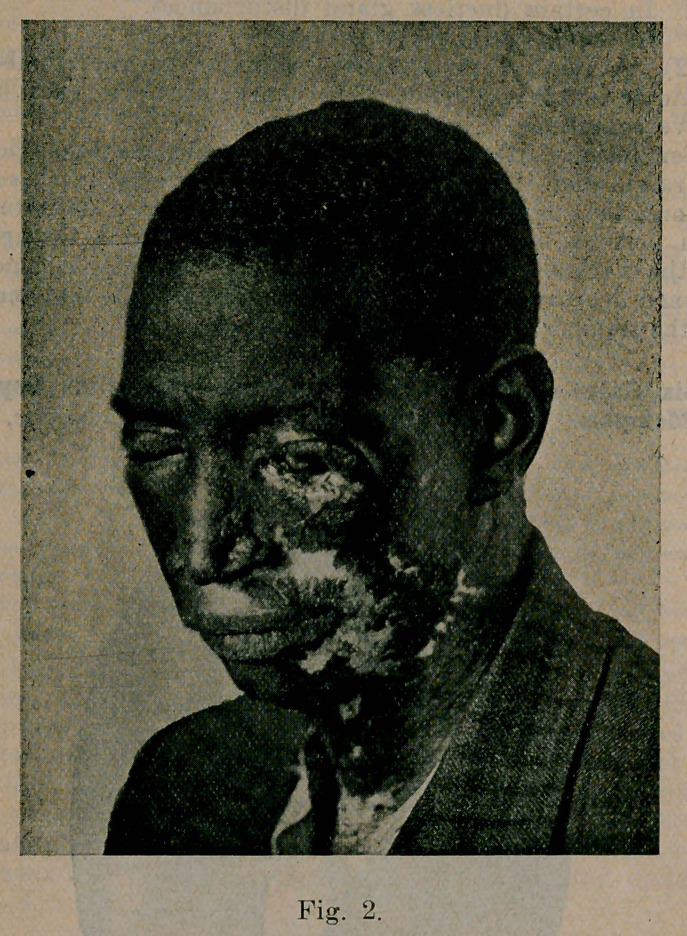


**Fig. 3. f3:**
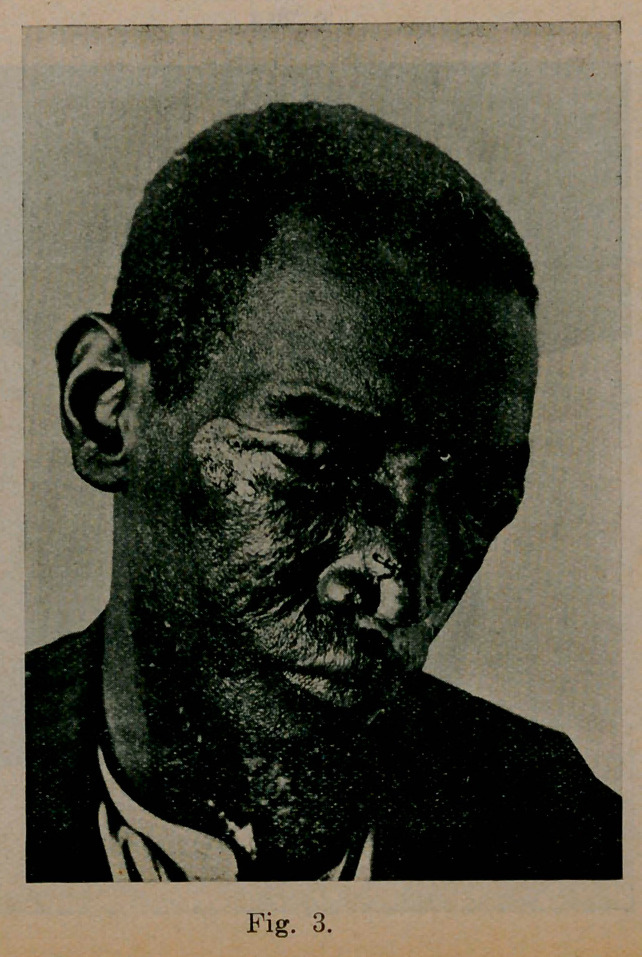


**Fig. 4. f4:**
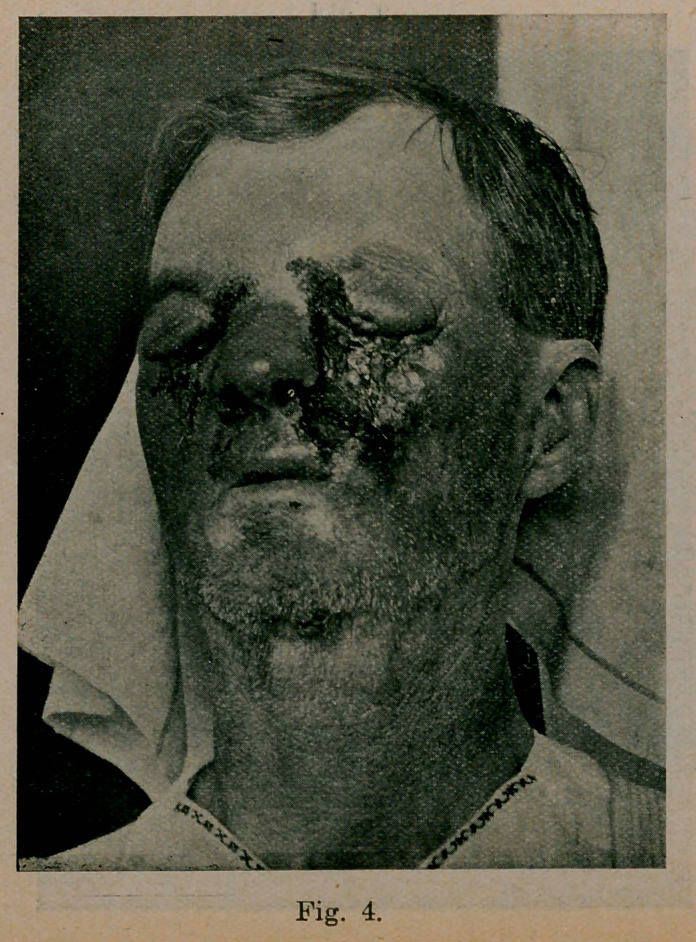


**Fig. 5. f5:**
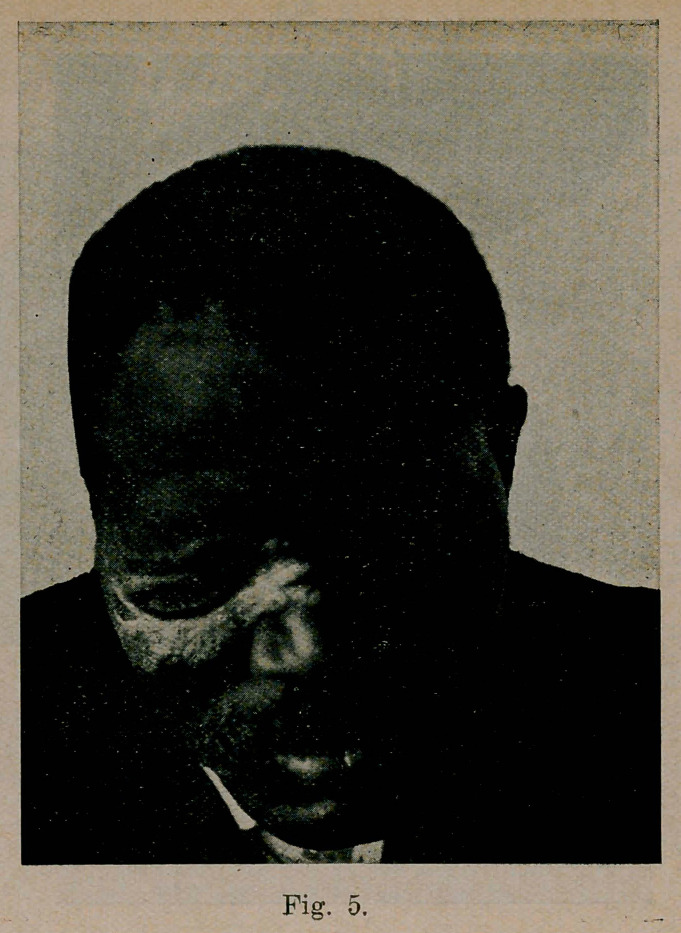


**Fig. 6. f6:**
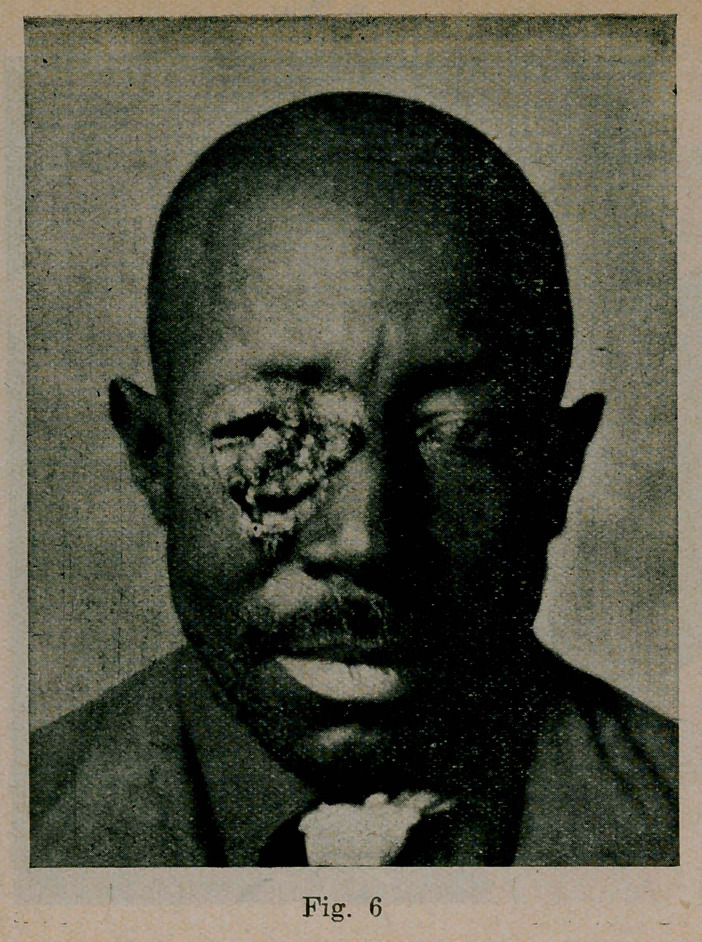


**Fig. 7. f7:**